# Supplementary material for: A qualitative exploration of consumers’ views and experiences toward online pharmacies: Narrative from a developing country
Source: PLoS One. 2025 Oct 10;20(10):e0331237. doi: 10.1371/journal.pone.0331237 (PMC12513602; doi:10.1371/journal.pone.0331237)
Supplement: Attachment — Submitted filename: Reviewers_comments_auresp_2.docx [file pone.0331237.s004.docx]

**Dear Editor and Reviewers,**

Thank you for taking the time to provide such detailed and helpful comments on our manuscript. We appreciate your constructive criticisms and suggestions, and we believe they will significantly improve the quality of our manuscript. We have revised the manuscript based on your feedback and would like to provide the following responses to your concerns.

**Reviewer #2**

Dear Authors,

Good job on making improvements to your manuscript. However, some minor and major adjustments are required to improve the quality and clarity of the writing. The following sections describe the required revisions.

**Minor revisions**

The manuscript requires extensive proofreading for grammatical accuracy.
1. Include "increase" in line 71.
2. Add "in" before Africa, Asia in line 106. Remove "India" from the statement, as India is part of Asia. Add "regions" after Latin America.
3. Add "es" after business in line 111. Remove "s" after strive.
4. Either remove "from" in line 451 or add "sources" after online.
5. Rewrite the sentence that begins in line 461 for clarity.
6. Rewrite the sentence that begins in line 468 and ends in line 470 for clarity.
7. Add a sentence or two in line 456 justifying why older and more educated people tend to prefer e-pharmacies. You can copy and rephrase from lines 93-96, but ensure that there is a statement on the education side of the argument.
8. "Preference for" is more appropriate than "diversion towards" in line 465, as people could still use physical stores while preferring e-pharmacies.
9. Medicine shortage "in physical stores" in line 466.
10. The sentence beginning in line 471 is not necessary where it is. Authors should either expunge or insert it in a more appropriate section.
11. The sentence beginning in line 491 can also be expunged as the paragraph could end without it.
12. Incomplete word "interne" in line 502.
Replace 'involved in' with "used for" in line 504.
13. Replace "of" in line 511 with "for".
14. Replace "life" with "lives" in line 512.
15. Replace "safe" with "safeguard" in line 521

**Response:** Thank you for comments and highlighting typo/grammatical errors. The manuscript is editing for English language by native English speaker to avoid any errors and confusion for the readers. Additionally, text on the older and educated people tends to prefer e-pharmacies is also added to the manuscript as suggested.

**Major revisions**

**Comment:** Contents in the limitation section do not appear like limitations. What are the actual limitations of the research? For instance, are there biases relating to group dynamics during data collection, e.g., dominant voices, social desirability bias, shallow personal narratives? Are there any limitations based on transcription and/or analysis? Are there any limitations that may arise using grounded theory, i.e., challenges with achieving theoretical saturation or researcher bias?

**Response:** Thank you for comments, the limitation section is rewritten again as per suggestions.

**Comment:** The practical implications section should be rewritten, as there are no specific practical implications mentioned. Also, the authors should specify and separate practical implications from theoretical, methodological, and policy implications.

**Response:** Thank you for comments, the practical implications section is rewritten again as per suggestions.

Thank you for your time and valuable feedback. We believe that the revisions have significantly improved the manuscript and hope that it is now suitable for publication.

Regard’s

**Corresponding author**
