# Supplementary material for: A qualitative exploration of consumers’ views and experiences toward online pharmacies: Narrative from a developing country
Source: PLoS One. 2025 Oct 10;20(10):e0331237. doi: 10.1371/journal.pone.0331237 (PMC12513602; doi:10.1371/journal.pone.0331237)
Supplement: Attachment — Submitted filename: Rebuttal to comments.docx [file pone.0331237.s005.docx]

**Dear Editor,**

Thank you for taking the time to provide such detailed and helpful comments on our manuscript. We appreciate your constructive criticisms and suggestions, and we believe they will significantly improve the quality of our manuscript. We have revised the manuscript based on your feedback and would like to provide the following responses to your concerns.

**In previous revision I got comments, and I addressed the comments as below:**

**Comment from Editor:**

**Comment 1:** Thank you for your responses; these have satisfactorily addressed the comments. **Response:** We appreciate your feedback and are pleased that our previous responses have addressed the earlier concerns.

**Comment 2:** I note that some of the participant quotes have been edited.

**Response:** We confirm that the quotes presented in the manuscript reflect the original responses provided by the participants, we did English editing for the that but as suggested in the comment below were reverted to its original quotes/wordings and are mentioned in the result section of manuscript.

**Comment 3:** As these are quotes, the wording should be provided by respondents, even if not grammatically correct.

**Response:** While minor grammatical and language edits were initially applied to enhance readability, these have now been removed in accordance with your recommendation.

**Comment 4:** Modifications should be indicated.

**Response:** No modifications remain in the current version. All quotes are now presented as quotes provided by the respondents without making any changes.

**However, in additional comments, the responses to these are below:**

**Comment 1:** Please amend your Response to Reviewers letter to include a point-by-point response to each of the points made by the Editor and / or Reviewers. Please follow this link for more information: <http://blogs.PLOS.org/everyone/2011/05/10/how-to-submit-your-revised-manuscript/>

**Response:** Thank you for comment, the rebuttal/response to comments by editor are done point to point in above text as suggested. Previously the response was in form of a paragraph but now it is point-by-point as per editors’ recommendations.

**Comment 2:** Please amend the title either on the online submission form or in your so that they are identical.

**Response:** Thank you for commenting and highlighting this issue, the title has been amended and made uniform in both the manuscript file and the editorial manager.

Thank you for your time and valuable feedback. We believe that the revisions have significantly improved the manuscript and hope that it is now suitable for publication.

Regard’s

**Corresponding author**
